# Induction and Monitoring of Adoptive Delayed-Type Hypersensitivity in Rats

**DOI:** 10.3791/325

**Published:** 2007-10-01

**Authors:** Christine Beeton, K. George Chandy

**Affiliations:** Department of Physiology and Biophysics, University of California, Irvine (UCI)

## Abstract

Delayed type hypersensitivity (DTH) is an inflammatory reaction mediated by CCR7- effector memory T lymphocytes that infiltrate the site of injection of an antigen against which the immune system has been primed. The inflammatory reaction is characterized by redness and swelling of the site of antigenic challenge. It is a convenient model to determine the in vivo efficacy of immunosuppressants. Cutaneous DTH can be induced either by adoptive transfer of antigen-specific T lymphocytes or by active immunization with an antigen, and subsequent intradermal challenge with the antigen to induce the inflammatory reaction in a given skin area. DTH responses can be induced to various antigens, for example ovalbumin, tuberculin, tetanus toxoid, or keyhole limpet hemocyanin. Such reactions can also be induced against autoantigen, for example to myelin basic protein (MBP) in rats with experimental autoimmune encephalomyelitis induced with MBP, an animal model for multiple sclerosis (1). Here we demonstrate how to induce an adoptive DTH reaction in Lewis rats. We will first stimulate ovalbumin-specific T cells in vitro and inject these activated cells intraperitoneally to naive rats. After allowing the cells to equilibrate in vivo for 2 days, we will challenge the rats with ovalbumin in the pinna of one ear, while the other ear wil receive saline. The inflammatory reaction will be visible 3-72 hours later and ear thickness will be measured as an indication of DTH severity.

**Figure:**
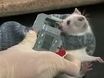


## Protocol

### 1. Stimulation of the ovalbumin-specific T lymphocytes

Resting ovalbumin-specific T cells are stimulated with ovalbumin in the presence of irradiated Lewis rat thymocytes (30 Gy) as antigen presenting cells (APCs), in Stimulation Medium (Prepare in DMEM + Pen/Strep/L-Glut + NEAA + RPMI Vitamins + 2ME + Rat Serum + Ovalbumin, at the final concentrations noted in the materials list.)

#### Rat serum preparation

1. We take serum from the rats we sacrifice as thymus donors.
2. Rats are deeply anesthetized by halothane inhalation and as much blood as possible is drawn by cardiac perfusion (5-10 ml per rat) using 10 ml syringes and 23 G 1" needles, after which the rats are immediately decapitated to ensure death.
3. The blood is allowed to clot for 10 min at 37°C followed by 10 min on ice.
4. The clot is removed and the remaining liquid spun.
5. The serum is collected, sterile-filtered and stored at -20°C.

#### Preparation of the antigen presenting cells

1. Take the thymus of a decapitated rat using sterile instruments and place it in PBS containing penicillin and streptomycin (PBS-PS), on ice.
2. Take the thymus to a tissue culture hood, clean it from blood clots and other contaminating tissue and cut it in small pieces into a cell strainer (Fisher # 08-771-2) placed in a 10 cm petri dish containing 10 ml of PBS-PS.
3. Each piece is pressed through the cell strainer using the back of a sterile 1 ml syringe plunger. Collect the single cell suspension into a 50 ml tube on ice.
4. Rinse the cell strainer with 20-40 more ml of PBS-PS. Centrifuge the suspension at 1250g and resuspend the pellet in PBS-PS, 5 ml/thymus, on ice.
5. Irradiate this suspension (30 Gy) in a gamma irradiator and immediately wash it twice with 50 ml PBS-PS.*Note: The best thymus donors are young adult rats, 5-7 weeks old. Rats up to 10 weeks may be used but the number of cells decreases with time. Gender is not important although some people say that males are better thymus donors. We always use females because all our animals are housed in the same room and we don't want males and females in the same room.*

#### Stimulation

1. Wash the T lymphocytes once and count them.
2. Count the antigen-presenting cells.
3. Mix 3 x 10^6^ ovalbumin-specific T cells with 150 x 10^6^ APCs in a 10 cm petri dish containing 10 ml of stimulation medium.
4. Incubate for 48 hours at 37°C.

### 2. Adoptive transfer of the ovalbumin-specific T lymphocytes

1. The rats we use as recipients are females 7-10 weeks old.
2. Count the T lymphocytes, they should be large and round. The antigen-presenting cells will be dead.
3. Centrifuge the T lymphocytes and resuspend them at 10 x 10^6^ cells/ml in PBS.
4. Inject 1 ml intraperitoneally using 3 ml syringes and 23 G 1" needles.

### 3. Challenge with antigen in the ear

1. Two days after adoptive transfer, inject 20 μl of a 1 mg/ml ovalbumin solution (= relevant antigen) in one ear and saline (or irrelevant antigen) in the other ear. Do NOT filter the antigen solution as you may lose aggregates that enhance the inflammatory reaction.*Note: The rats should be anesthetized to avoid movements. If you are right-handed, hold the ear between your left thumb and index finger, the index finger being inside the ear and the rat facing you.*
2. Inject the solution in the pinna of the ear using 27G ½" needles. Try to inject all the rats exactly at the same place.

### 4. Measurement of DTH

1. The ear will start turning red and swell as a sign of inflammation. Measure the thickness of each ear with a spring-loaded micrometer (for example model PK-0505 from Mitutoyo).
2. Take 5-6 measurements for each ear at each time-point and calculate the average.
3. The maximum swelling should occur between 24 and 72 hours.

## Discussion

The antigenic challenge can also be performed at other sites, for example in the skin of the back (2). We however find that challenging. Using the pinna of the ear, as shown in this video, allows for the most precise measurement of the DTH reaction.
